# Dynamics and Patterning of 5-Hydroxytryptamine 2 Subtype Receptors in JC Polyomavirus Entry

**DOI:** 10.3390/v14122597

**Published:** 2022-11-22

**Authors:** Kashif Mehmood, Michael P. Wilczek, Jeanne K. DuShane, Matthew T. Parent, Colleen L. Mayberry, Jaqulin N. Wallace, Francois L. Levasseur, Tristan M. Fong, Samuel T. Hess, Melissa S. Maginnis

**Affiliations:** 1 Department of Molecular and Biomedical Sciences, The University of Maine, Orono, ME 04469, USA; 2 Department of Physics & Astronomy, The University of Maine, Orono, ME 04469, USA; 3 Graduate School of Biomedical Science and Engineering, The University of Maine, Orono, ME 04469, USA

**Keywords:** JC polyomavirus, 5-HT_2_ receptors, receptor clustering, super resolution microscopy, FPALM

## Abstract

The organization and dynamics of plasma membrane receptors are a critical link in virus-receptor interactions, which finetune signaling efficiency and determine cellular responses during infection. Characterizing the mechanisms responsible for the active rearrangement and clustering of receptors may aid in developing novel strategies for the therapeutic treatment of viruses. Virus-receptor interactions are poorly understood at the nanoscale, yet they present an attractive target for the design of drugs and for the illumination of viral infection and pathogenesis. This study utilizes super-resolution microscopy and related techniques, which surpass traditional microscopy resolution limitations, to provide both a spatial and temporal assessment of the interactions of human JC polyomavirus (JCPyV) with 5-hydroxytrypamine 2 receptors (5-HT_2_Rs) subtypes during viral entry. JCPyV causes asymptomatic kidney infection in the majority of the population and can cause fatal brain disease, and progressive multifocal leukoencephalopathy (PML), in immunocompromised individuals. Using Fluorescence Photoactivation Localization Microscopy (FPALM), the colocalization of JCPyV with 5-HT_2_ receptor subtypes (5-HT_2A_, 5-HT_2B_, and 5-HT_2C_) during viral attachment and viral entry was analyzed. JCPyV was found to significantly enhance the clustering of 5-HT_2_ receptors during entry. Cluster analysis of infected cells reveals changes in 5-HT_2_ receptor cluster attributes, and radial distribution function (RDF) analyses suggest a significant increase in the aggregation of JCPyV particles colocalized with 5-HT_2_ receptor clusters in JCPyV-infected samples. These findings provide novel insights into receptor patterning during viral entry and highlight improved technologies for the future development of therapies for JCPyV infection as well as therapies for diseases involving 5-HT_2_ receptors.

## 1. Introduction

The plasma membrane serves as the initial site of response generation between living cells and various external signals. External ligands engage membrane-localized receptors, which bind to specific target stimuli and generate signaling cascades across the membrane into the cell that will decide the fate of cells in response to the stimulus [1]. Receptors cluster in nanoscopic domains on the plasma membrane where they control ligand sensitivity to improve efficiency and protein interaction [2]. Examples include G-protein coupled receptors (GPCRs) [3], immune-cell receptors [1], as well as receptors hijacked by microbial toxins [4] or viruses [5]. During the past couple of decades, research has emphasized the importance of the spatial localization of receptors such as GPCRs in their response to specific signaling pathways crucial for physiological pathways [6,7]. Furthermore, as human diseases are correlated to aberrations in the distribution of membrane-bound receptors and/or their activation, it is important to characterize and understand the mechanisms underlying the dynamic rearrangement and clustering of receptors, as it may aid in developing novel strategies for therapeutic treatment of diseases [8]. Recent work has established super-resolution microscopy as a useful technique for the nanoscale study of cellular receptors, including the formation of homo- and hetero-oligomers and more extended clusters [9,10].

Advancements in microscopy have been pivotal for biological studies, especially in addressing membrane organization and ligand-receptor interactions. These advancements include groundbreaking localization-based super-resolution microscopy techniques; fluorescence photoactivation localization microscopy (FPALM) [11], stochastic optical reconstruction microscopy (STORM) [12], and photoactivated localization microscopy (PALM) [13], which utilize the photochemical properties of specific fluorescent probes by stochastically activating sparse subsets of them, imaging those visible molecules, and determining their locations from their diffraction-limited images [11]. Ultra-resolution structures are then generated by rendering the images of multiple fluorophores localized over time [11,14]. Thus, FPALM and similar techniques can be used to map biological components and their dynamics at the nanoscale level, helping to provide a more resolved and accurate representation of critical biological events.

In the past two decades, super-resolution microscopy techniques have been employed in a variety of virological studies, lending new perspectives with improved levels of confirmation. For example, super-resolution microscopy has led to insights into the redistribution of envelope proteins in human immunodeficiency virus (HIV) virion maturation [15] and specific recruitment of HIV envelope proteins to assembly sites for virion formation [16]; the assembly of the replication complex by yellow fever viruses [17]; the identification of the dual function of CD81 in influenza virus uncoating and budding processes [18]; the organization of hepatitis C virus (HCV) structural proteins in lipid droplets [19]; the tracking of adenovirus genomes in infected host cells [20]; the mapping of viral architecture in vaccinia virus [21] and the visualization of morphological changes in the endoplasmic reticulum during Zika virus infection [22]. Further, FPALM imaging has been utilized in conformational studies of biological structures such as actin at the nanoscale [23]; the organization of caveolin-1 below the diffraction limit in a living vertebrate animal model [24], and the modulation of clustering of the cellular lipid phosphatidylinositol 4,5-bisphosphate (PIP2) in the presence of influenza hemagglutinin [25]. These advances using super-resolution microscopy have provided a deeper understanding of biological events and provide the groundwork for further discovery in the field of virus-host cell interactions. Considering the size of viruses and the scale of viral–host cell interactions [14], the diffraction limit of conventional microscopy techniques hampers the identification of these events [26]. Yet, super-resolution studies of virus–receptor interactions have so far been limited. Currently, there is little knowledge about the rearrangements of host-cell receptors in the context of JCPyV infection. Therefore, in the present study, we employed super-resolution microscopy to bridge this gap to understand how JC polyomavirus (JCPyV) alters the dynamics of host-cell serotonin 5-hydoxytryptamine (5-HT_2_) receptors in the context of JCPyV infection.

JCPyV is the causative agent of progressive multifocal leukoencephalopathy (PML), a viral disease that is rare but often becomes debilitating and fatal due to the lysis of glial cells in the central nervous system (CNS) [27,28,29]. Although PML is a rare disease, nearly 80% of adults worldwide are seropositive for JCPyV [30,31,32,33,34]. In the majority of cases, JCPyV causes an asymptomatic infection in the kidney [32,35,36,37]. In immunocompromised individuals such as patients with HIV/AIDS or those undergoing immunomodulatory treatments, the infection can spread within the CNS, infecting and lysing oligodendrocytes and astrocytes, eventually causing PML [38,39,40]. Due to the successful use of combination antiretroviral therapy (cART) treatments, the occurrence of PML in individuals with HIV/AIDS has dropped (~0.1%) compared to the prevalence of PML in ~5–8% of HIV patients prior to cART [41]. Although rare, PML can also occur in people with hematological malignancies, such as lymphoma and leukemia. Additionally, PML incidence has risen in the past two decades in those receiving prolonged immunomodulatory therapies such as natalizumab, rituximab, and efalizumab [42,43,44,45,46]. The incidence of PML in these patient populations led to the withdrawal of natalizumab and efalizumab from the market, yet natalizumab was later reintroduced with significant safety warnings and risk stratification [41,47]. Despite the significant reduction in incidence rate in the past two decades, the survival rate of up to 1 year for PML remains at ~75% in natalizumab-related PML patients [48] and ~70% in HIV-related patients under treatment [49,50,51,52,53]. In addition, the limited treatments available typically involve restoring the immune system which can cause other fatal CNS complications such as immune reconstitution inflammatory syndrome [49,54,55]. Therefore, it is vital to understand how JCPyV establishes infection in human cells.

Human JCPyV is a member of the viral *Polyomaviridae* family, characterized by non-enveloped virions containing a double-stranded DNA genome [56,57]. The JCPyV capsid is approximately 40 nm in diameter and is comprised of three viral capsid proteins: viral protein 1 (VP1), VP2, and VP3 [58,59]. JCPyV initiates infection either through extracellular vesicles [60] or by directly binding to α2,6-linked sialic acid receptors [61,62,63] and/or non-sialylated glycosaminoglycans (GAGs) on host cells via the VP1 protein [64]. Subsequently, the virus enters the cell through the utilization of the 5-hydroxytryptamine 2 (5-HT_2_) serotonin receptors [65,66]. Among the different isoforms of 5-HT receptors, only the subtype 2 receptors, 5-HT_2A_R, 5-HT_2B_R, and 5-HT_2C_R, support JCPyV entry by clathrin-mediated endocytosis [67]. A transient interaction has been observed between JCPyV and each of the 5-HT_2_R subtypes at 5 min post-infection [68]. However, the underlying mechanism and spatial distribution of 5-HT_2_ receptors during JCPyV entry and infection remains unclear and hindered by microscopy resolution limits [66].

Expressed in the kidneys [69,70] and CNS, 5-HT_2_ receptors are classified as GPCRs, which mediate a large array of physiological and behavioral functions in humans, including mood regulation and social cognition [71], neurogenesis [72], memory [73], and depression [74]. GPCRs comprise 14 different isoforms [75], and specifically, 5-HT_2_ receptors respond to ligand stimuli by desensitization using receptor internalization via clathrin-mediated endocytosis [76,77]. Upon agonist activation, GPCRs can go through substantial reorganization from their base levels, including cluster formation (as in μ opioid receptors [78]) or modulation of receptor diffusion kinetics (as in metabotropic glutamate receptor mGluR3 [79]), which changes the redistribution of receptors in clathrin-coated pits [80]. Variability in patient response to antidepressant drugs was shown to depend on changes in the clustering of serotonin transporters as well as 5-HT_2A_ receptors [81]. While spatial reorganization and clustering of GPCRs can mediate their function [3], not enough is known about the clustering of 5-HT_2_ receptors and their nanoscale distribution and dynamics in living cells, which are important for treatments.

5-HT_2_ receptors are necessary for a crucial step in the infectious viral lifecycle of JCPyV, facilitating entry into host cells. Still, the clustering and dynamics of the receptors that facilitate viral entry are unknown. This study exploits FPALM to provide crucial insight into spatial and temporal interactions between JCPyV and the 5-HT_2_ receptors to define how these interactions promote viral infection at the nanoscale. Our results indicate that viral localization varies between the 5-HT_2_ receptor subtypes necessary for viral internalization at different time points postinfection. These data also reveal the cluster properties of 5-HT_2_ receptors in response to the virus, furthering our understanding of the pattern and dynamics of host cell receptors in the presence of JCPyV and providing a novel view of virus–host interactions. Additionally, these findings demonstrate the cluster dynamics of 5-HT_2_ receptors in response to ligands, including neurotransmitters and target therapeutics, and provide new information about GPCRs [82]. Altogether, this research enhances our knowledge of viral entry and understanding of JCPyV invasion of host cells, which could inform the development of potential antiviral therapies for PML.

## 2. Materials and Methods

### 2.1. Cells, Viruses, Antibodies, and Reagents

HEK293A cells were grown in Dulbecco’s minimal essential medium (DMEM, Corning, Corning, NY, USA) with 10% fetal bovine serum (FBS), 1% penicillin-streptomycin (P/S, Mediatech, Inc., Manassas, VA, USA) and 0.2% plasmocin prophylactic (InvivoGen, San Diego, CA, USA). HEK293A stable cells expressing 5-HT_2_R subtypes (5-HT_2A_, 5-HT_2B,_ and 5-HT_2C_) in fusion with YFP [67] were maintained in DMEM with, 10% FBS, 1% P/S, 0.2% plasmocin prophylactic (InvivoGen), and 500 µg of G418 (Corning). Cell lines were graciously provided by the Atwood laboratory (Brown University, Providence, RI, USA). All cell types were cultured at 37 °C with 5% CO_2_ in a humidified incubator.

JCPyV strain Mad-1/SVEΔ was kindly provided by Atwood laboratory (Brown University). The generation of JCPyV was described previously [83,84] and the proliferation of JCPyV was performed using published protocols [85]. Alexa Fluor 647-labeled JCPyV (JCPyV-647) was prepared as described [63,67]. Viral titers were determined through fluorescent focus unit (FFU) infectivity assays in SVG-A cells [67]. Viral infectivity FFU assays were performed using antibodies derived from two hybridoma-derived supernatants, one containing a monoclonal antibody specific for JCPyV VP1 (PAB596) that can cross-react with SV40 VP1 (generously provided by Ed Harlow) and a monoclonal antibody that is exclusively specific against JCPyV large T antigen (PAB962) (generously provided by Tevethia laboratory, Penn State University, State College, PA, USA). Additionally, an anti-pan cadherin antibody (1:200, Abcam) and DAPI nuclear counter strain (1:1000, Thermo Fisher Scientific, Waltham, MA, USA) was used for confocal immunofluorescence imaging.

### 2.2. Generation of 5-HT_2_R-Dendra2 Fusion Constructs

The constructs for 5-HT_2A_R-YFP, 5-HT_2B_R-YFP, 5-HT_2C_R-YFP, and pEYFP-N1 (Clontech, Mountain View, CA, USA) were graciously provided by Atwood laboratory (Brown University). To generate the 5-HT_2A_R-Dendra2 constructs, both the 5-HT_2A_R-YFP and HA-Dendra2 [86] constructs were double digested with BamHI (NEB) and NotI-HF (NEB). The digested products were gel eluted on a 1% agarose gel (Invitrogen) using a silica bead-based DNA gel extraction kit (Thermo Scientific, Waltham, MA, USA) according to the manufacturer’s protocol. The gel-purified Dendra2 gene and 5-HT_2A_R containing pEYFP-plasmid (without YFP gene) were ligated to generate a C-terminal fusion of the 5-HT_2A_ receptor to the Dendra2 gene. The empty Dendra2 construct was generated in the same manner using BamHI and NotI enzymes to replace the YFP gene with the Dendra2 gene in the pEYFP plasmid. To generate the 5-HT_2B_R-Dendra2 fusion construct, a cDNA fragment of the 5-HT_2B_R gene was PCR amplified with a 40 nucleotide-5′ primer containing a HindIII site followed by a Kozak sequence and the first 25 nucleotides specific to 5-HT_2B_R open reading frame (ORF) (Table 1) and a 3′ primer complementary sequence to the last 23 nucleotides of the ORF with a BamHI site in place of the stop codon (Table 1). Similarly, for generating the 5-HT_2C_R-Dendra2 and Dopamine Receptor D2 (DRD2)-Dendra2 constructs, cDNA for both genes were amplified using individual 5′ primers with an XhoI site followed by a Kozak sequence and 20 nucleotides specific to the start sequence for each ORF, respectively, and 3′ primers complementary sequences of 26 nucleotides and 24 nucleotides, respectively, followed by a BamHI site replacing the stop codon. In the 5′ primers of DRD2 cDNA, a degenerate mutation was introduced at the 2nd codon (T→C) to remove the BamHI site. The PCR cDNA products of all three 5-HT_2B_R, 5-HT_2C_R, and DRD2 ORFs were purified using a silica bead DNA extraction kit (Thermo Scientific), double digested along with the empty Dendra2 plasmid for each ORF using a respective pair of restriction enzymes and gel extracted using a 1% agarose gel (Invitrogen) and a silica bead DNA extraction kit (Thermo Scientific). The gel-eluted products of each gene were ligated with the respective Dendra2 plasmids that were digested with the same pair of restriction enzymes, such that the Dendra2 gene is fused to the C-terminus of the appropriate receptor. All plasmids were sequence verified for orientation and confirmed using a CMV-F universal forward primer and a Dendra2-N reverse primer (Table 1) at The University of Maine DNA sequencing facility and analyzed using FinchTV (Version 1.4.0) software.

### 2.3. Generation of HEK293A Stable Cell Lines for 5-HT_2_R-Dendra2

HEK293A cells plated in a T-25 flask (25 cm^2^-Cell Star) up to 80% confluence were transfected with 13 µg of 5-HT_2A_R-, 5-HT_2B_R-, 5-HT_2C_R–Dendra2, and DRD2-Dendra2 fusion or empty Dendra2 constructs using Fugene HD transfection reagent (Promega) according to the manufacturers’ instructions. The cells were incubated at 37 °C for 48 h in DMEM containing 10% FBS, 1% P/S (Mediatech, Inc., Manassas, VA, USA), and 0.2% plasmocin prophylactic (InvivoGen). The medium was then replaced with selective media, consisting of DMEM containing 10% FBS, 1% P/S (Mediatech, Inc.), 0.2% plasmocin prophylactic (InvivoGen) and 500 µg of Geneticin G418 (Corning) to select for cells transfected with the Dendra2 constructs containing the Geneticin resistance cassette. The medium was then replaced every 3 days for 1 week. Subsequently, the cells were expanded in a T-75 flask (75-cm^2^ Cell Star) under selective pressure for an additional week and confirmed for percent transfection and viability. Cells were maintained under a selective medium and the expression of constructs was verified by epifluorescence microscopy at the start and through the duration of each experiment.

### 2.4. Indirect Immunofluorescence Detection and Quantification of JCPyV Infection

HEK293A stable 5-HT_2A_R-Dendra2, 5-HT_2B_R-Dendra2, 5-HT_2C_R-Dendra2, and DRD2-Dendra2 cells were plated to 70% confluency in 96-well plates and infected with JCPyV at a multiplicity of infection (MOI) of 2 FFU/cell at 37 °C for 1 h and then replenished with complete DMEM and incubated at 37 °C. At 48 h postinfection, cells were washed with 1× phosphate buffer saline (PBS) and fixed using 4% paraformaldehyde (PFA) at room temperature (RT) for 10 min. After fixation, stable cells were permeabilized with 1× TBS-1% Triton X-100 for 15 min and blocked in 20% goat serum (Vector Labs, Newark, CA, USA) in 1× PBS. Following permeabilization, cells were washed and stained with PAB962 (T-antigen Ab, 1:2) at 37 °C for 1 h. Cells were then washed with 1× PBS and stained with an anti-mouse Alexa Fluor 633 secondary antibody (Thermo Fisher) at 37 °C for 1 h and stained for nuclei using DAPI stain (Thermo Fisher) at RT for 5 min. Virus infectivity was quantified by counting the number of T-antigen positive cells per five visual fields (per well, per sample), using a Nikon Eclipse Ti epifluorescence microscope (Micro Video Instruments, Inc., Avon, MA, USA). Infectivity was determined by dividing the number of infected cells per visual field by the total number of DAPI-positive nuclei per visual field as previously described [87,88]. The average percent infection was normalized to the control sample 5-HT_2A_R-YFP (100%) [66,89]. Data were plotted as a bar graph faceted by the three 5-HT subtypes and the negative control using R (version 4.0.5) using the package ggplot2 [90].

### 2.5. Cell-Surface Expression of 5-HT_2_R-Dendra2 Fusion Proteins in HEK293A Stable Cells

5-HT_2_R-Dendra2 fusion protein-expressing HEK293A stable cells were plated to 60% confluency in a 6 mm 96 wells (#1.5H) glass bottom plate (CellVis) along with control samples in complete DMEM with 500 µg of G418 (Corning). At 24 h post-plating, cells were washed with 1× PBS and fixed using 4% PFA (Invitrogen) for 10 min and washed again three times with 1× PBS. After washing, cells were incubated with blocking buffer (2% goat serum, 0.2% Triton X-100 and 0.1% BSA in 1× PBS) at RT for 1 h and then stained with an anti-pan-cadherin antibody (1:200, Abcam) in blocking buffer t at 4 °C O/N. Cells were washed three times with 1× PBS and stained with a secondary anti-mouse Alexa Fluor-647 antibody (1:1000) in blocking reagent at RT for 1 h. Furthermore, cells were washed and stained for nuclear marker using DAPI counterstain (1:1000) at RT for 5 min. After the final washes, cells were observed by confocal microscopy using an Olympus laser scanning confocal microscope (model IX81; Olympus America, Inc., Westborough, MA, USA) at 60× magnification (oil immersion) using FluoView software (version 04.01.01.05). Fields of view were visualized through z-sectioning using the DAPI channel, and fluorescence images were obtained for cell nuclei (blue), receptor/control (green) and anti-pan cadherin (magenta) channels using 405/635-, 543/488-nm multiline argon laser, and a 633 HeNe laser, respectively. Cell-surface expression of receptors was determined using ImageJ by defining Manders’ overlap coefficient utilizing colocalization threshold analysis (FIJI) [91]. Each field of view in comparable z-planes was analyzed and represents the percentage of overlap between the pan-cadherin cell-surface marker and Dendra2/YFP expression for expressed receptors. Data were plotted in a box graph and statistics were calculated using GraphPad Prism (GraphPad Software, Inc., San Diego, CA, USA).

### 2.6. Analysis of JCPyV Attachment in Stable Cells

5-HT_2_R-Dendra2 fusion protein-expressing HEK293A stable cells were plated in 12-well plates and were removed upon 100% confluency using CellStripper (Corning, Corning, NY, USA), followed by centrifugation at 414× *g* at 4 °C for 5 min to make pellets. Cells were washed with 1× PBS, pelleted again, and resuspended in 10% complete phenol-free MEM (Corning). Cells were incubated on the ice at 4 °C for 45 min. Cells were pelleted and resuspended in 200 μL of 10% complete phenol-free MEM containing Alexa Fluor 647-labeled JCPyV and were further incubated on ice at 4 °C for 1 h to permit viral attachment. Cells were pelleted, washed with 1× PBS, fixed in 4% PFA for 10 min, and then resuspended in 300 μL of 1× PBS. For the analysis of viral attachment in fixed cells, flow cytometry was carried out by using a BD FACSCanto instrument (BD Biosciences) equipped with a 633-nm AP-C laser line (Becton, Dickinson, and Company, Franklin Lakes, NJ, USA) for 10,000 events before analysis with BD FACSDiva (Becton, Dickinson, and Company) and FlowJo software (TreeStar, Inc., Ashland, OR, USA). To exclude complex and dead cells from the samples, FlowJo Software was used to gate the samples.

### 2.7. Quantification of JCPyV Entry in Stable Cells

Cells seeded in a 96-well, #1.5 glass-bottomed plate (CellVis) to ~70% confluence were chilled on ice at 4 °C for 45 min prior to infection. Cells were then incubated with Alexa Fluor 647-labeled JCPyV (HEK293A cells) in MEM containing 2% FBS and 1% P/S on ice at 4 °C for 1 h for viral attachment. To allow for viral entry, the cells were further incubated at 37 °C for 1.5 h. Subsequently, the cells were washed in 1× PBS and fixed in 4% PFA for 10 min. Using 60× objective (oil immersion), cells with Dendra2 expression were viewed to define the field of view, and images were taken for differential interference contrast (DIC), Dendra2, and JCPyV-647 expression in respective channels. Viral entry was quantified by making masks in ImageJ software and generating regions of interest (ROIs) (adjusted to remove plasma membrane of cells) for at least 30 Dendra2-expressing cells in each sample type, which were further used to measure the mean fluorescent units of JCPyV-647 per cell in a background-corrected sample within an applied threshold intensity. Cross-sections were measured for at least three independent experiments.

### 2.8. Preparing Samples for Two-Color FPALM Experiment

HEK293A stable cells were plated up to 60% confluency in three separate 6 mm, 96-well (#1.5H) glass bottom plates (CellVis) along with control samples in complete DMEM with 500 µg of G418 (Corning). After 24 h, plates were incubated at 4 °C for 1 h to chill the cells, then cells were infected with JCPyV-647 at an MOI of 3 FFU/cell. Plates were incubated at 37 °C and fixed at 0-, 5-, and 15-min post-infection (mpi) using 4% PFA at RT for 10 min. Fixed plates were washed three times with 1× PBS before processing for imaging.

### 2.9. Super-Resolution Fluorescence Photoactivation Localization Microscopy (FPALM)

Samples were processed and imaged as described previously [92,93]. In brief, the experimental setup was performed using an Olympus (Tokyo, Japan) IX71 inverted microscope with 60X 1.45 NA total internal reflection fluorescence (TIRF) microscopy objective lens, 2X telescope in the detection path, and an Andor iXon + electron-multiplying charge-coupled device (DU897-DCS-#BV; Andor, Belfast, UK). Excitation was achieved by employing a 558 nm laser (CrystaLaser, Reno, NV, USA) and activation using 405 nm (FBB-405- 050-FSFS-100; RGBlase LLC, Fremont, CA, USA) in either widefield or TIRF illumination. The corresponding lasers were aligned together with mirrors and a dichroic (Z405RDC; Chroma, Rockingham, VT, USA) and directed into an f = 350 mm convex lens (Thorlabs, Newton, NJ, USA) at one focal length from the objective back aperture plane. FPALM image acquisition was carried out with a frame rate of 30–50 Hz and an electron multiplication gain of 200. For Dendra2 single-channel data acquisition, the detection filters consisted of both a T565LP (Chroma) or a 561RU dichroic (Semrock, Rochester, NY, USA), a 405 nm and two 561 nm notch filters (NF03-405E-25 and NF561E-25; Semrock), and a 605/70 emission filter (Chroma). Raw data were then processed and analyzed with custom MATLAB software (The MathWorks, Natick, MA, USA). For the determination of parameters, including fluorescent molecule locations, a nonlinear least-squares algorithm was used for fitting PSFs to two-dimensional Gaussians (an approximation of the PSF). Acquired camera frames were processed according to standard algorithms for the identification and localization of individual emitters [86].

### 2.10. Glucose Oxidase Imaging Buffer

For imaging, the organic dye Alexa 647, a glucose oxidase buffer (GLOX) was needed to allow the organic dye to blink for super-resolution imaging. GLOX was prepared on the day of imaging based on the protocol for dSTORM [12,94] and according to the following reagent concentrations: 10 µg/mL Catalase from bovine liver (Sigma-Aldrich, C9322, St. Louis, MO, USA), 50 µg/mL glucose oxidase from Aspergillus niger (Sigma-Aldrich, G2133), 10 mM Tris-HCL, 10 mM MEA, and 10% glucose diluted into PBS. All reagents were prepared individually and combined only just prior to imaging. New GLOX buffer was made every hour of imaging, as the effects of the GLOX buffer were noticeably reduced after this time.

### 2.11. Multicolor Widefield FPALM Imaging of JCPyV and Mock Infected Samples

The experimental protocol for image acquisition was performed as described previously [86,93]. Laser illumination alignment setup included 405 and 558 nm lasers along with far-red 638 lasers (CrystaLaser, Reno, NV, USA) for JCPyV-647 treated samples and the illumination trail was always co-aligned with all the beams. The sets of filters and dichroic employed in the current setup comprised the following: dichroic in the microscope turret, DiO1R 405/488/561/635 (Semrock); notch filters in detection path (2× 405 nm, 2× 561 nm, and a 635 nm; all Semrock); dichroic in the two-color detection module, FF580-FDi01 (Semrock); emission filter in the red channel, a Brightline 664 LP (Semrock), and in the orange channel, and FF-01 585/40 (Semrock). Fluorescent beads (Tetraspeck microspheres, 0.1 mm; Invitrogen (Carlsbad, CA, USA)/ThermoFisher Scientific (Waltham, MA, USA)) were used for spatial orientation and calibration during image acquisition in both target channels concurrently. The final images were also double-checked systematically by using a previously described algorithm [86] to determine the transformation that best aligned the two channels. After background subtraction using a temporal median filter [95], the localization of fluorophores was performed with a pixel intensity threshold (typically 12–20 photons) for the merged pair of channels or using separate thresholds for each channel when one label was significantly brighter than the other (as for Alexa-647 and Dendra2). Localizations were scrutinized for a good fit, the number of photons per localization (within the range of 10–3000 photons), point spread function width (1/*e*^2^ radius 140–600 nm), localization uncertainty (<80 nm), and error in fitted point spread function width (<150 nm) to exclude failed or poor fits. Surface localizations crossing the tolerance threshold were drift corrected, and individual species were calculated using cutoffs for α set as the ratio of intensities of each localized spot in the red channel over the total intensity (red plus orange channels) [93]. The cutoffs were determined using the measured α histograms for cells labeled with only one of the two species and imaged on the same day. Typically, Dendra2 was spotted as α < 0.79, and Alexa-647 was identified as α > 0.80. Bleedthrough rates for this combination of probes were also estimated from the α histograms to be 0.3% (average for Dendra2 and Alexa Fluro-647) and <1% for Dendra2 and Alexa Fluro-647. When calculating RDFs and Manders’ Coefficients, bleedthrough correction was applied using the method from Dahan Kim et al., 2013. Individual molecules are binned into a small grid (20 nm) and then the number count is corrected in each pixel according to equation:

$$n_{A}^{Corr}=\frac{n_{A}^{Meas}-k_{BA}n_{A}^{Meas}-k_{BA}n_{B}^{Meas}}{1-k_{AB}-k_{BA}}$$

$$n_{B}^{Corr}=\frac{n_{B}^{Meas}-k_{AB}n_{A}^{Meas}-k_{AB}n_{B}^{Meas}}{1-k_{AB}-k_{BA}}$$

where $n_{A}^{Meas}$ and $n_{B}^{Meas}$ are the binned “intensities” (number counts of molecules of different types) before bleed-through correction, $n_{A}^{Corr}$ and $n_{B}^{Corr}$ are the bleedthrough corrected values, and $k_{AB}$ and $k_{BA}$ are the rates of bleed-through from channel A into channel B and channel B to channel A, respectively. For each experiment, the magnification of the microscope was determined by using the image of a standard calibrated scale and measured using ImageJ. The calculation of the pixel-value-to-photon conversion factor was conducted using previously published methods [23].

## 3. Results

### 3.1. 5-HT_2_R-Dendra2 Forms Clusters on the Cell Surface of Stably Transfected Cells

To better define receptor clustering and the role of 5-HT_2_Rs in JCPyV infection, localization-based super-resolution microscopy was employed to visualize 5-HT_2_Rs at the plasma membrane. Plasmid constructs that express 5-HT_2_R subtypes tagged with the photoactivatable fluorescent protein Dendra2 [96] (5-HT_2A_R-Dendra2, 5-HT_2B_R-Dendra2, and 5-HT_2C_R-Dendra2) and, for comparison, the dopamine receptor tagged with Dendra2 (DRD2) were created for use in FPALM (Figure S1). DRD2 was selected because it is also a GPCR but is not required by JCPyV for entry or infection [65]. Furthermore, the expression of 5-HT_2A_R, 5-HT_2B_R, and 5-HT_2C_R in poorly-permissive HEK293A cells restores infection [66]. Thus, to generate stable cell lines, 5-HT_2_R-Dendra2 constructs were transfected into HEK293A cells and sustained in antibiotic-selective media, as shown in the schematic representation (Figure S1) and 5-HT_2_R-Dendra2 construct-expressing cells were confirmed for restoration of JCPyV infection (Figure S2).

**Figure 1:**
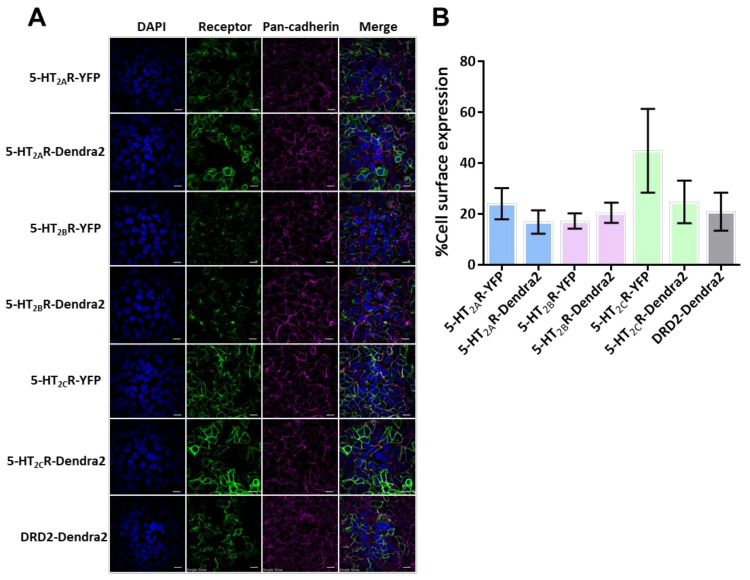
Cell-surface characterization of 5-HT_2_R-Dendra2 protein compared to 5-HT_2_R-YFP in stable cell lines. (**A**) Representative images of stably-expressing 5-HT_2_Rs-YFP, 5-HT_2_Rs-Dendra2, and DRD2- Dendra2 constructs (green) in HEK293A cells, by confocal microscopy using a 60× objective lens (1.45 NA). Pan-cadherin (cell-surface marker) expression is shown in magenta, receptor in green and DAPI (nuclei) in blue. Scale bar = 10 µm. (**B**) Cell-surface expression levels for 5-HT_2_R subtypes and DRD2-Dendra2 compared to 5-HT_2_R subtypes-YFP were measured. Analysis was performed by quantifying Manders’ colocalization coefficient by measuring the percent colocalization of receptor expression with the pan-cadherin antibody (as surface marker) using ImageJ software. Percent cell-surface expression is averaged from at least 10 fields of view in three independent experiments. Error bars = SEM.

To confirm cell-surface expression, the stable cell lines were plated, fixed after 24 h and stained for the cell membrane marker pan-cadherin [67]. Cell-surface expression of receptors was confirmed and measured in stable HEK293A cells expressing 5-HT_2_Rs (5-HT_2A_, 5-HT_2B,_ and 5-HT_2C_) and DRD2 using confocal microscopy and image analysis (Figure 1A). Expression levels of 5-HT_2_Rs and DRD2 stable cells were compared to previously published 5-HT_2A_R-YFP, 5-HT_2B_R-YFP, and 5-HT_2C_R-YFP stable cells that support an increase in JCPyV entry and infection compared to control HEK293A cells [66,67,89]. The stable cells demonstrated a distinct cell-surface expression in all 5-HT_2_R sample types and the DRD2 sample (Figure 1A). Images were analyzed to quantify the cell-surface expression of each receptor by measuring the percent overlap between the fluorescence intensities of the receptor and the stain for the cell-surface cadherins using ImageJ [67] (Figure 1B). The expression levels of each serotonin receptor are similar to the comparable 5-HT_2_R-YFP stable cell lines (Figure 1B). Altogether, the Dendra-5-HT_2_R-expressing cells represent a model system in which each 5-HT_2_R receptor type is fluorescently labeled and present at the cell surface.

### 3.2. Cell-Surface Characterization of 5-HT_2_Rs Using FPALM-TIRF Microscopy

**Figure 2:**
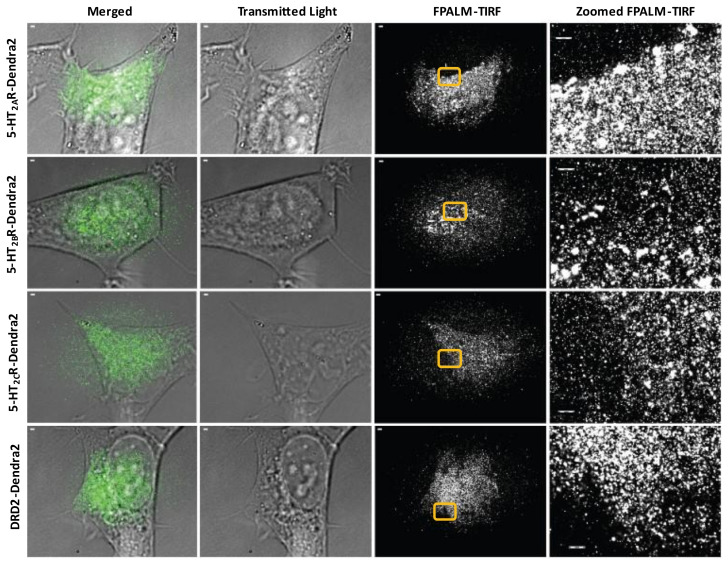
Surface expression of 5-HT_2_R-Dendra2 fusion proteins using FPALM-TIRF microscopy. HEK293A stable cells expressing 5-HT_2A_R-Dendra2, 5-HT_2B_R-Dendra2, 5-HT_2C_R-Dendra2, and DRD2-Dendra2 (green) were analyzed using super-resolution localization microscopy (FPALM) with TIRF illumination (individually represented by each row). Columns represent images acquired using different channels, with column of merged images on the left followed by column of transmitted light images and column of FPALM-TIRF images for each sample. Rightmost column represents zoomed-in regions of interest (yellow box) in FPALM-TIRF images for individual samples. Images were obtained using an Olympus IX71 with 60× 1.45 NA TIRFM objective, excitation with a 561 nm laser, and activation with a 405 nm laser. Raw image stacks were acquired with an Andor iXon EMCCD at a frame rate of 31 Hz and EM gain of 200. Scale bars = 1 µm.

Proper expression of membrane-bound receptors is vital to the utilization of the receptors by ligands and downstream events [97] including viral infection [98]. To define the spatial distribution of 5-HT_2_Rs in HEK293A cells, FPALM-TIRF microscopy was employed to visualize and characterize the Dendra2-fused receptors on the plasma membrane (Figure 2). Cell lines stably expressing receptors were plated on 96-well glass-bottom dishes and fixed 24 h after plating. FPALM-TIRF imaging of the Dendra2-expressing 5-HT_2_Rs revealed a clustered distribution of receptors on the plasma membrane for each 5-HT_2_R subtype (5-HT_2A_, 5-HT_2B_, and 5-HT_2C_) (Figure 2). The observed distribution in the plasma membrane suggests that the receptors are (a) present on the cell surface and (b) clustered in a manner similar to that of other cell membrane-bound receptors viewed via TIRF microscopy in previous studies [99].

### 3.3. Manders’ Colocalization Analysis: 5-HT_2_Rs Colocalize with JCPyV during Virus Entry

**Figure 3:**
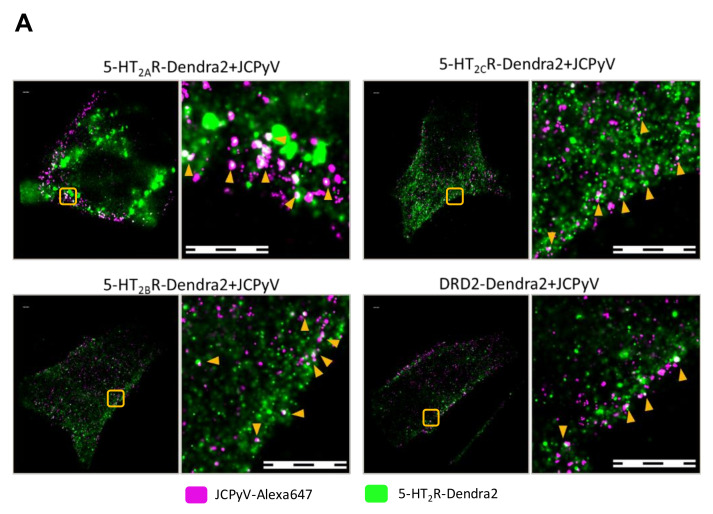

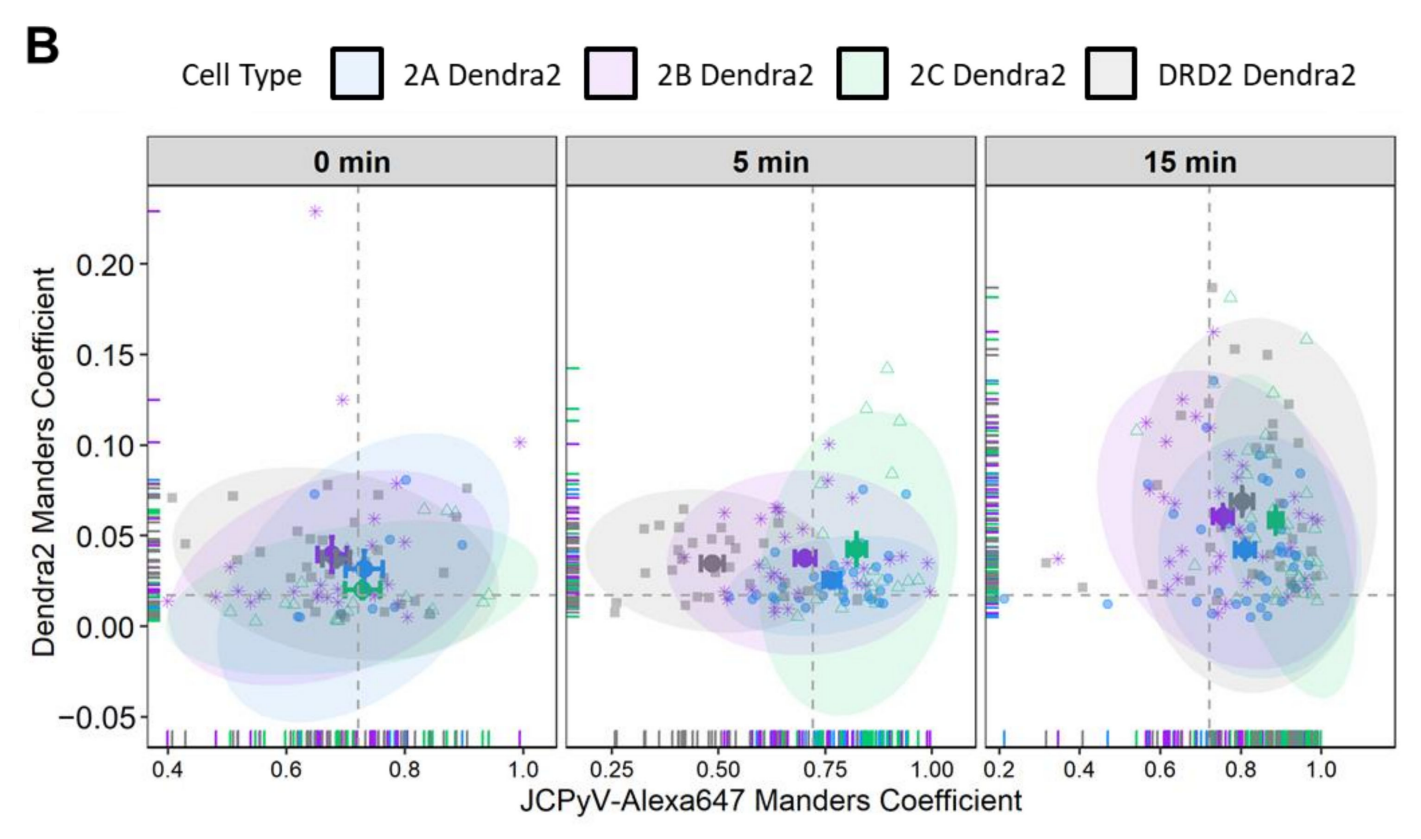
FPALM analysis demonstrates JCPyV localization with 5-HT_2_R-Dendra2 and DRD2- Dendra2. (**A**) Representative widefield super resolution FPALM images of 5-HT_2A_R-Dendra2, 5-HT_2B_R- Dendra2, 5-HT_2C_R-Dendra2, and DRD2-Dendra2 at 5 min post infection (mpi). Scale bars = 1 µm. (**B**) Manders’ colocalization coefficients (MCC) demonstrated by scatter plot showing analysis for JCPyV-647 incubated with corresponding 5-HT_2_Rs-Dendra2 and DRD2-Dendra2 stable cells at 0, 5, and 15 mpi. Samples were excited using 561 nm and 638 nm lasers, and activated using a 405 nm laser, and imaged with a 60× 1.45NA TIRF objective. Normal distribution of the population determined by Shapiro Wilks test and Quantile-Quantile plot. Significance was determined using the pairwise Wilcox test and the Bonferroni method was used for the *p*-value adjustment. Fluorescence images were pre-processed, and the background signal was removed. MCCs were calculated after rendering molecules by convolving localizations with a normalized 50 nm circle. Cell areas were masked using an automatic algorithm. Populations represent at least 10 cells per time point and sample; representative of three independent replicates. Error bars = SEM.

Previous studies and work from the Maginnis laboratory demonstrate that 5-HT_2_ receptors are not required for JCPyV attachment, yet they are essential for virus internalization [66,67]. Assetta et al. utilized a proximity ligation assay (PLA) to show transient interactions between JCPyV and 5-HT_2A_, 5-HT_2B_, and 5-HT_2C_ receptor subtypes at 5 min post-infection (mpi) [68]; however, no interactions were observed at 0 and 15 mpi, and the nanoscale distribution of these 5-HT receptors during entry and infection has not been studied. To further define the interactions between JCPyV and 5-HT_2_Rs, we used two-color super-resolution FPALM to image and analyze colocalization between Alexa647-labeled purified JCPyV (JCPyV-647) with 5-HT_2A_R-, 5-HT_2B_R-, and 5-HT_2C_R-Dendra2 fusion proteins, DRD2, and the Dendra2-only expressing stable cell lines at 0, 5, and 15 mpi (Figure 3). DRD2 and Dendra2 only were used for comparison as they did not support JCPyV infection (Figure S2). Colocalization of JCPyV with all the 5-HT_2_Rs was observed across all three time points (Figure 3B, Table 1). Quantification of the colocalization events was evaluated using Manders’ Coefficient of Colocalization (MCC) between the Dendra2-tagged receptor and JCPyV-647, for all three timepoints, with at least three independent experiments of at least ten cells each (i.e., a total of ≥30 cells for each timepoint) (Figure 3B, Table 1). These results demonstrate JCPyV colocalization with 5-HT_2_R-Dendra2 for all three subtypes, 5-HT_2A_R, 5-HT_2B_R, and 5-HT_2C_R, as well as with the DRD2 receptor through all three time points (Figure 3B, Table 1)), even though DRD2 receptors are not required for viral infection (Figure S2) [65]. JCPyV colocalization with receptors varies with different time points postinfection; however, significant differences were only observed between the virus and Dendra2-tagged 5-HT_2_R subtypes and the DRD2 receptor at 5 mpi (Figure 3B). At 0 mpi, JCPyV-647 colocalizes with 5-HT_2_Rs (5-HT_2A_, 5-HT_2B_, and 5-HT_2C_) as well as DRD2, and no significant difference was observed between them (Figure 3B, Table 1 and Table 2).

At 5 mpi, a significant increase (58% ± 6%; *p* < 0.001) was observed in JCPyV-647 colocalization with 5-HT_2A_R-Dendra2 when compared to JCPyV-647 colocalization with DRD2, with significance determined using a Wilcoxon Rank Sum Test (Table 1). Similarly, a significant increase of 45% ± 6% (*p* < 0.001) in JCPyV-647 colocalization with 5-HT_2B_R-Dendra2 and a 69% ± 6% (*p* < 0.001) increase in JCPyV-647 colocalization with 5-HT_2C_R were observed in comparison to JCPyV-647 colocalization with DRD2 (Figure 3B, Table 1 and Table 2). At the 15 mpi, similar levels of colocalization were observed in JCPyV-647 with 5-HT_2_R subtypes compared to DRD2; however, among 5-HT_2_Rs, there was a significant increase (9%± 3%; *p* < 0.05) in JCPyV-647 colocalization with 5-HT_2C_R compared to colocalization with 5-HT_2A_R, and a 17%± 3% increase in JCPyV-647 colocalization with 5-HT_2C_R compared to JCPyV-647 colocalization with 5-HT_2B_R (*p* < 0.001) (Figure 3B, Table 1 and Table 2). Cumulatively, the Manders’ colocalization analysis demonstrates that JCPyV colocalizes with 5-HT_2_R subtypes as well as the DRD2 receptor at all three timepoints, but there is a significant increase in JCPyV colocalization with 5-HT_2_R subtypes at 5 mpi when compared to the DRD2 receptor (Table 1 and Table 2).

### 3.4. JCPyV Attachment Is Not Enhanced in Cells Expressing 5-HT_2_ Receptors

**Figure 4:**
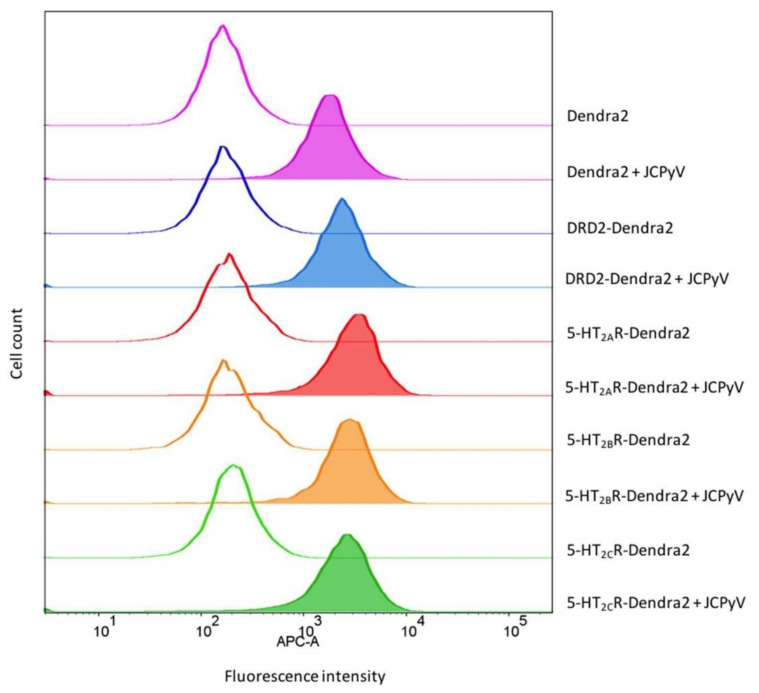
JCPyV attachment is not enhanced in 5-HT_2_R-expressing cells. Indicated stable cell lines were incubated with JCPyV-647 (filled histograms) or mock-treated (open histograms) on ice for 1 h and analyzed by flow cytometry. Histograms represent a comparison of JCPyV-647 attachment to HEK293A cells stably expressing 5-HT_2A_R-Dendra2 (red), 5-HT_2B_R-Dendra2 (orange), 5-HT_2C_R-Dendra2 (green), DRD2-Dendra2 (blue), and Dendra2 (purple) proteins constructs. Histograms represent the mean fluorescence intensities for mock-treated (unfilled), or samples treated with JCPyV-647 (filled). Data are representative of 10,000 events for individual samples representative of 3 independent experiments. Data were analyzed using FlowJo software.

JCPyV binds to host cells through α2,6-linked sialic acid receptor motifs and/or non-sialylated glycosaminoglycans (GAGs) via the attachment protein VP1 [62,63,64,100], and internalization is mediated by 5-HT_2_ receptors [65,66]. Previous research has demonstrated that viral attachment to host cells remains unaffected upon the expression of 5-HT_2_ receptors when compared to control cells [66], suggesting that attachment and entry constitute a two-step process. A JCPyV infectivity assay using cell lines stably expressing 5-HT_2_Rs, DRD2, or Dendra2 showed a significant increase in JCPyV infectivity of 5-HT_2_Rs-expressing cells compared to DRD2- and Dendra2-expressing cells (Figure S2), confirming previous findings [66]. To test whether stable cell lines expressing Dendra2-tagged 5-HT_2_ receptors show enhanced JCPyV attachment, flow cytometry was used to quantify viral attachment as a function of receptor subtype. Stable cells expressing 5-HT_2_Rs, DRD2, or Dendra2 were plated, incubated for 24 h, removed from plates, and incubated with JCPyV-647 on ice for 1 h. JCPyV-647 attachment was measured in virus-treated and mock samples using flow cytometry (Figure 4). Equivalent levels of JCPyV-647 mean fluorescence intensities were observed in cells expressing 5-HT_2_Rs, DRD2, and Dendra2, suggesting that JCPyV attachment is not significantly affected by overexpression of 5-HT_2_Rs or DRD2. This confirms that the stable cells do not enhance or reduce viral attachment to host cells, and these data are consistent with previously-reported results with 5-HT_2_R-YFP [66].

### 3.5. JCPyV Entry Is Enhanced in 5-HT_2_R-Expressing Cells

**Figure 5:**
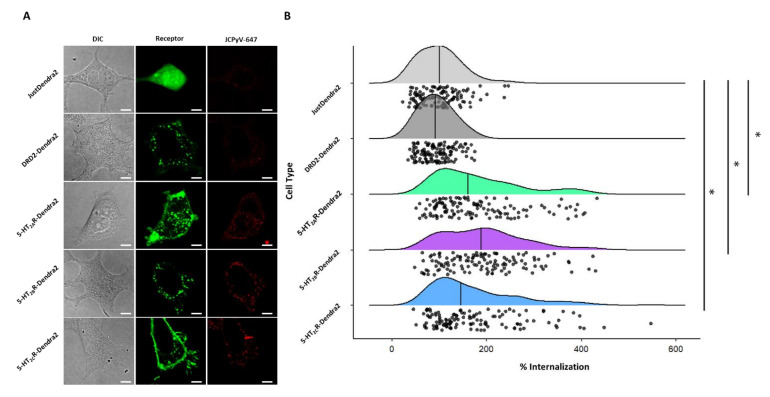
JCPyV internalization significantly increases in 5-HT_2_R subtype-expressing stable cells. Cells stably expressing 5-HT_2_R subtypes were plated and infected with JCPyV-647 (MOI = 3 FFU/cell) on ice for synchronized virus attachment and further incubated at 37 °C for 1.5 h to allow for viral entry. After fixation, cells were visualized via confocal microscopy using a 60× objective lens. (**A**) Images demonstrate DIC (grey), Dendra2 expression (green), and JCPyV-647 (red) in individual samples. Using Image J, masks were drawn for Dendra2- expressing cells in individual samples and JCPyV internalization was quantified using the relative fluorescence intensity of JCPyV-647 within individual cells for at least 30 cells of each sample per replicate for 3 independent replicates. (**B**) Quantified data are shown as a raincloud plot with individual data points indicated in black and the distribution shown with the median indicated by a black line. The Wilcoxon Rank Sum Test was used to compare the percent internalization of each cell type. The percent internalization of 5-HT_2A_R-Dendra2, 5-HT_2B_R-Dendra2, and 5-HT_2C_R-Dendra2 subtypes was significantly different compared to DRD2-Dendra2 and JustDendra2. There was no significant difference between percent internalization of JCPyV between 5-HT_2_R subtypes. The symbol * signifies *p* < 0.01. Scale bars = 5 μm.

Previous studies have established that JCPyV infection is increased when 5-HT_2_Rs are overexpressed in semi-permissible cells, such as HEK293A cells [66] and Figure S2. Further, the 5-HT_2_R-mediated increase in JCPyV infection is attributed to a significant enhancement of viral internalization [65,66,67]. To determine whether the expression of Dendra-tagged 5-HT_2_Rs or DRD2 increases the internalization of JCPyV in stable cells, viral entry was measured using confocal microscopy. Stable cell lines expressing Dendra2-tagged 5-HT_2_Rs, DRD2, or Dendra2 were plated and incubated with JCPyV-647 on ice to synchronize viral attachment, then further incubated at 37 °C for 1.5 h for viral entry. Cells were fixed and viral internalization was measured by confocal microscopy. The quantification of the relative fluorescence intensity of JCPyV-647 within individual cells was determined using ImageJ [67,101]. Representative confocal images of individual samples for viral internalization (Figure 5A) demonstrate an increase in JCPyV-647 fluorescence in 5-HT_2_R-expressing samples compared to DRD2 and Dendra2 samples. Quantification was performed to measure internalized JCPyV-647 by generating cell masks in ImageJ, and then by measuring fluorescence intensities inside individual cells, excluding the cell membrane (Figure 5B) (similar to [101] but with the mask generation performed manually). Stable cells expressing 5-HT_2_Rs demonstrated a significant increase in viral internalization when compared to DRD2- and Dendra2-expressing stable cells. Taken together, these data demonstrate that the stable cell lines expressing Dendra2-tagged 5-HT_2_Rs support and enhance JCPyV internalization with equivalent efficiency. These data further suggest that while JCPyV localizes in membrane areas where DRD2 is expressed (Figure 3, Table 1), DRD2 does not enhance viral entry [66].

### 3.6. JCPyV Changes Cluster Properties for 5-HT_2_ Receptor Subtypes in Infected Cells

The clustering of receptors at the plasma membrane and interactions between receptors and ligands results in the translation of exogenous signals into a cellular response [102]. Previous studies suggested that viral attachment to cell-surface receptors involved in endocytosis would cause spatial confinement of virus particles immediately following attachment [2]. Recent advancements in microscopic techniques have improved the analysis of spatial arrangements and patterning of cell membrane receptors and suggest that aggregation of receptor clusters can induce functional consequences that are not predictable from individual components [1].

To characterize the spatial dynamics and aggregation of 5-HT_2_ receptors in the presence of JCPyV, widefield FPALM images obtained were analyzed and quantified for cluster properties of receptors in 5-HT_2_Rs, DRD2, and Dendra2 in JCPyV-infected cells at three timepoints (0, 5, and 15 mpi). The Dendra2 sample was used as a negative control for receptor cluster densities. Individual clusters were identified using both the radial distribution function and single linkage cluster analysis, and the physical properties of clusters were analyzed for cluster density, circularity, and area [103].

**Figure 6:**
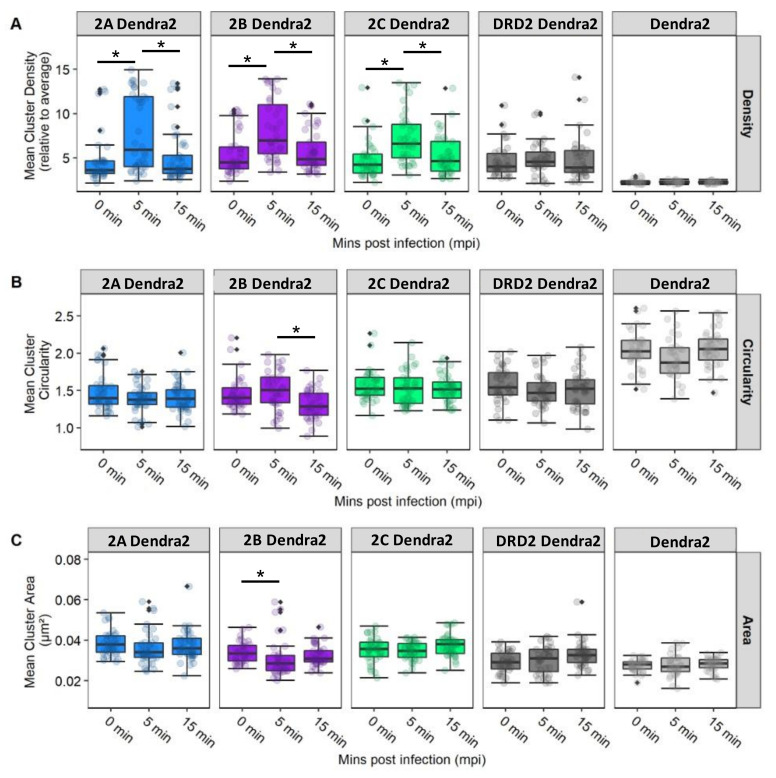
5-HT_2_R cluster properties for receptors subtypes in infected cells. Cluster properties including (**A**) mean cluster density, (**B**) circularity, and (**C**) area were calculated for localizations of 5-HT_2_R-Dendra2 subtypes, DRD2-Dendra2 (Dopamine Dendra2), and Dendra2 in stable cells fixed at 0, 5, and 15 mpi and analyzed by widefield FPALM. Clusters were identified by binning molecules onto a grid and convolving with a circle (radius 50 nm) and thresholding for regions with density ≥3 times the cell average. The columns represent sample conditions, and rows represent the physical characteristics of clusters for each sample. In individual graphs, the y-axis indicates the value for each cluster characteristic, while the x-axis represents the 3 different time points. Cluster density is significantly increased at 5 mpi in cell types that support JCPyV infection. The Kruskal–Wallis test was used to compare the intensity values of each cluster property comparing all timepoints within each cell type. The symbol * indicates *p* < 0.05. Outliers are represented by black diamonds.

Figure 6 illustrates the cluster properties of 5-HT_2_R subtypes and DRD2 in cells infected and imaged at three timepoints post-infection (data shown are the average of three independent replicates with at least 10 images per sample per replicate). Comparing individual cluster properties for each sample across the three timepoints post-infection, a significant (68% ± 12%; *p* < 0.05) increase was observed in the median cluster density of 5-HT_2A_R from 0 to 5 mpi, and a subsequent decrease in density of (−36% ± 12%; *p* < 0.05) was observed from the 5 min to the 15 min timepoint (Figure 6). Similarly, an increase in median cluster density by 52% ± 10% (*p* < 0.05) was observed for 5-HT_2B_R from 0 to 5 mpi, and a decrease in density of −30% ± 9% (*p* < 0.05) was observed from 5 mpi to 15 mpi. The trend was also observed in the 5-HT_2C_R sample, with an increase of 49% ± 10% (*p* < 0.05) in median cluster density at 5 min compared to 0 mpi, while a decrease of −28% ± 10% (*p* < 0.05) was observed in mean cluster density from 5 to 15 mpi. Significance was determined using a Kruskal–Wallis test. No such significant change in median cluster density was observed between the three time points for the control DRD2 and Dendra2 samples. Considering cluster area, the only significant change was observed in the 5-HT_2B_R sample, in which the median cluster area decreased −9% ± 5% (*p* < 0.05) from 0 mpi to 5 mpi. Furthermore, a significant change in cluster circularity was only observed in the 5-HT_2B_R sample, with median cluster circularity decreasing −12% ± 3% (*p* < 0.05) from the 5 min to the 15 min timepoint. Collectively, these data represent a significant increase in median cluster densities of 5-HT_2_ receptor subtypes from 0 mpi to 5 mpi, and a subsequent decrease in those same densities from 5 mpi to 15 mpi. This significant increase in median receptor-cluster density was only observed in cells stably expressing Dendra2-tagged 5-HT_2_Rs and was not observed in the DRD2 or control (Dendra2) samples. Our data demonstrate that JCPyV changes the cluster properties of 5-HT_2_ receptors in the plasma membrane of host cells during viral attachment and entry, suggesting that alterations in 5-HT_2_ receptor patterning are associated with JCPyV entry.

### 3.7. Radial Distribution Function Analysis: JCPyV Aggregation within and Adjacent to 5-HT_2_ Receptor Clusters

**Figure 7:**
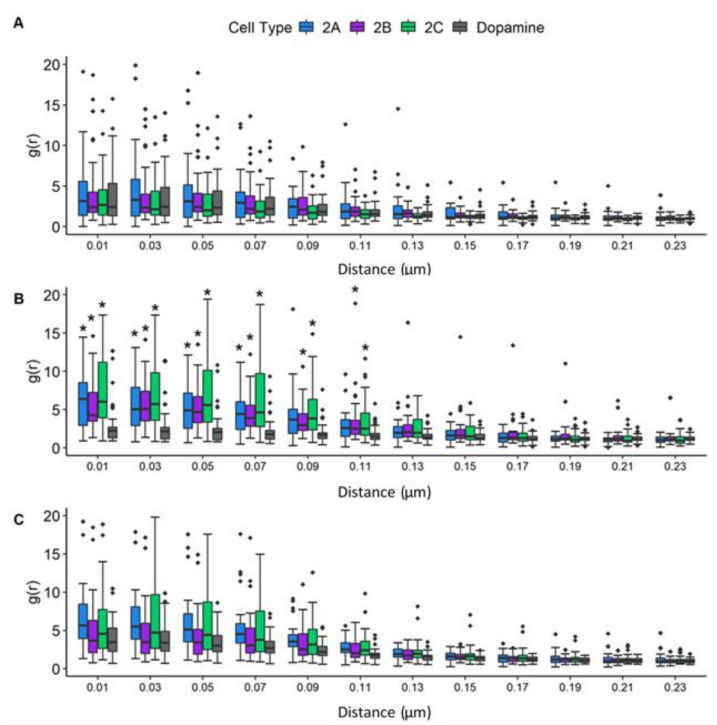
JCPyV aggregates significantly under 5-HT_2_R subtypes at 5 min post-infection. The radial distribution function (RDF) was analyzed to determine the spatial distribution of 5-HT_2_R-Dendra2, DRD2-Dendra2, and JustDendra2 (control) clusters at (**A**) 0, (**B**) 5, and (**C**) 15 mpi using widefield FPALM images. The Kruskal-Wallis test was used to compare the RDF values at each distance measured in each cell comparing all cell types across each timepoint. The symbol * indicates significant differences (*p* < 0.01). The RDF value (y-axis) denotes the density of molecules as a function of distance from the cluster center (r, x-axis), with an RDF value of 1 indicating the density expected for a random distribution. Populations represent ~30 cells per time point per sample from three independent experiments, representative of an average of three independent replicates. Outliers are represented by black diamonds.

Recent data highlight the importance of the characterization of spatial patterning of cell surface receptor signaling clusters, as their patterns can reveal aspects of their function which are invisible to non-imaging methods [1]. To characterize the spatial distribution of JCPyV localized in or adjacent to 5-HT_2_R receptor clusters of infected cells, the radial distribution functions (RDFs), denoted by amplitude (g) as a function of radius (r), were calculated for the identified clusters of 5-HT_2_R subtypes and DRD2 in JCPyV-infected cells, as well as for JCPyV-647 particles in the vicinity. JCPyV-647 RDF calculations were performed for infected cells expressing 5-HT_2_Rs and DRD2 at 0, 5, and 15 mpi (Figure 7). Each row represents JCPyV-RDFs for all samples at the given timepoint (i.e., Figure 7A represents JCPyV-RDFs at 0 min, Figure 7B represents JCPyV-RDFs at 5 min, and Figure 7C shows JCPyV-RDFs for all samples at 15 mpi). In individual graphs, the g(r) value (y-axis) indicates the probability of an average JCPyV being found at a given distance r (x-axis) from the center of the cluster, with g(r) = 1 expected for a random distribution of the virus. Results demonstrate the concentration of JCPyV molecules toward the center of a 5-HT_2_R cluster at all three timepoints (Figure 7). Significant changes in the RDFs of JCPyV particles within 5-HT_2_R or DRD2 receptor clusters were not observed at 0 mpi. However, at 5 mpi, a significant increase (*p* < 0.01 with the Kruskal–Wallis test) in the density of JCPyV particles adjacent to or within the receptor clusters was observed in JCPyV-RDFs when comparing infected samples expressing 5-HT_2_R-Dendra2 with infected samples expressing DRD2-Dendra2. No significant difference was observed for JCPyV-RDFs between infected cells expressing various 5-HT_2_R subtypes. Furthermore, no significant difference was observed between the JCPyV-RDFs comparing infected cells expressing 5-HT_2_Rs or DRD2 samples at 15 mpi (Figure 7). Altogether, these data show a significant increase in the relative density of JCPyV particles adjacent to or within 5-HT_2_Rs clusters in infected cells at 5 mpi when compared to infected cells expressing DRD2. These results demonstrate the enhanced aggregation of virus particles within 5HT_2_ receptor clusters at 5 mpi, which is a time consistent with the colocalization of JCPyV with clathrin and viral entry [67].

## 4. Discussion

**Figure 8:**
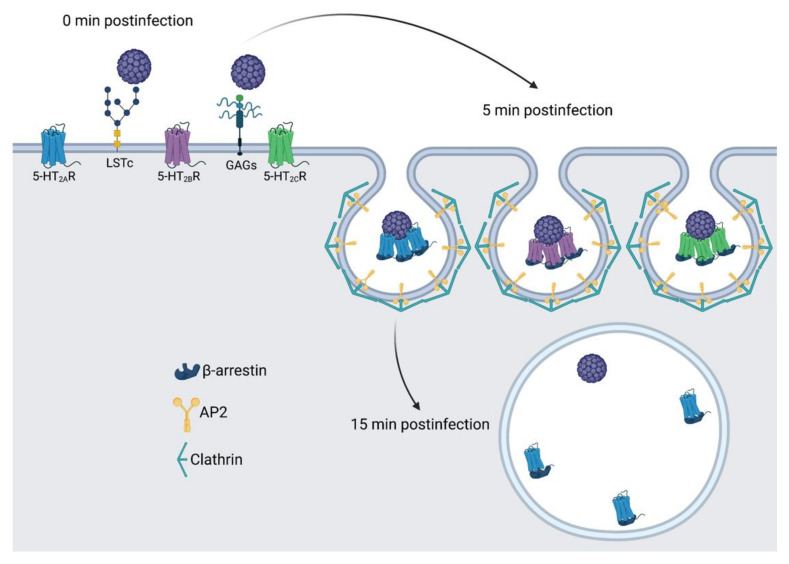
Model of 5-HT_2_R reorganization and clustering during JCPyV entry. Receptor-mediated infection of JCPyV is initiated by attaching to cell-surface receptors including sialic-acid containing LSTc or GAGs, shown at 0 mpi. At 5 mpi, 5-HT_2_Rs cluster and virions are localized to the center of the cluster, and this timing is consistent with viral entry via clathrin-mediated endocytosis. At 15 mpi, receptor clustering of 5-HT_2_Rs is not observed, and the virus may be deposited in an early endosome. Figure not to scale. Figure created using BioRender.

While previous work has detailed structures of viral proteins in complex with their receptors [104,105,106], gaps remain in our understanding of the dynamics of these interactions. Super-resolution microscopy techniques have enabled the nanoscale visualization of viruses and interactions with specific host cell receptors in living cells [14]. Plasma membrane expression and arrangement/organization of cell surface receptors in nanoscale domains are also crucial for proper induction and spatiotemporal control of signaling pathways associated with these receptors. GPCRs tend to generate clusters in nanoscopic domains, which are essential for adjusting ligand sensitivity, which controls protein interactions and signaling [3]. Previous studies have established that JCPyV can enter cells in a receptor-independent manner using extracellular vesicles [60] or in a receptor-dependent mechanism [66,67,68]. In a receptor-supported infection, JCPyV binds to α2,6-linked sialic acid receptors including lactoseries tetrasaccharide c (LSTc) [61,62,63] and/or non-sialylated glycosaminoglycans (GAGs) on host cells via the capsid protein VP1 [64] to initiate infection, and JCPyV then utilizes 5-HT_2_ receptors to enter the host cell [65,66,67]. However, it is yet to be demonstrated how JCPyV reorganizes 5-HT_2_ receptors to drive the internalization of virus particles. The present study was designed to observe the spatiotemporal patterning of 5-HT_2_ receptors in the presence of JCPyV, which can serve as a model for other virus-receptor or ligand-5-HT_2_ receptor interactions. Using super-resolution microscopy (FPALM) [11], our findings illuminate the dynamics of 5-HT_2_ receptors during virus internalization (Figure 8).

In this study, cells stably expressing 5-HT_2_R (5-HT_2A_R-Dendra2, 5-HT_2B_R-Dendra2_,_ and 5-HT_2C_R-Dendra2) subtypes resulted in increased JCPyV infectivity compared to dopamine-receptor-Dendra2 (DRD2) and control Dendra2-expressing cells (Figure S2), consistent with previously published results [66,67,89]. Furthermore, 5-HT_2_Rs labeled with Dendra2 (5-HT_2A_, 5-HT_2B,_ and 5-HT_2C_) were observed on the cell surface, and expression of 5-HT_2_ receptors did not impact JCPyV attachment but rather increased viral internalization compared to controls (Figure 4, Figure 5 and Figure 8), as expected [66,67]. Using super-resolution microscopy, we demonstrate the nanoscale colocalization of JCPyV with the three 5-HT_2_ receptor subtypes (5-HT_2A_, 5-HT_2B,_ and 5-HT_2C_) during the initial steps of viral attachment and viral entry (Figure 3), suggesting that the viral utilization of host receptors might be dependent on localization and clustering in the proximity of 5-HT_2_ receptors. Transient colocalization of JCPyV with 5-HT_2_ receptor subtypes has been previously reported using the proximity ligation assay [65,68]; however, our data further highlight the differences between JCPyV colocalization with individual 5-HT_2_ receptors and their nanoscale cluster formation at all three timepoints using super-resolution microscopy. Furthermore, the data presented herein demonstrate significant increases in the mean cluster density of 5-HT_2_ receptors in JCPyV-infected cells at 5 mpi (Figure 6) during which time JCPyV has been shown to induce endocytosis. Additionally, this study identified an increase in aggregation of JCPyV particles colocalized with the 5-HT_2_ receptor clusters at 5 mpi (Figure 7 and Figure 8). The findings presented enhance our understanding of cell-surface receptor cluster properties in response to viral infection and illuminate how receptor reorganization can influence critical steps in viral infection.

Data from FPALM images of infected 5-HT_2_-expressing cells show that JCPyV colocalizes with 5-HT_2_ receptors during the viral attachment and entry process. Even though JCPyV colocalization was observed at all three time points (0, 5, and 15 min) postinfection, the colocalization of JCPyV was observed to be significantly higher with 5-HT_2_ receptors compared to DRD2-receptors at 5 mpi (Figure 3). Published data strengthen our results showing higher colocalization of JCPyV with 5-HT_2_ receptors at 5 mpi [65,68]. These data suggest the persistent colocalization of JCPyV with 5-HT_2_ receptors through viral attachment and entry might cause changes in the clustering of 5-HT_2_ receptors.

In recent years, advancements in microscopy have proven to be vital for studying such nanoscopic domains of receptor clusters, providing deeper insight into signal initiation and transduction mechanisms [107,108,109]. Our data emphasize high-resolution details for virus–receptor studies which are currently limited. Previous findings demonstrated that some agonists could cause a redistribution of GPCRs in the plasma membrane, such as μ opioid receptor clusters upon activation with the ligand DAMGO, leading to receptor clustering followed by endocytosis [78,80]. Our data highlight that JCPyV induces clustering of 5-HT_2_ receptors at length scales inaccessible to diffraction-limited microscopy. Upon cluster analysis of infected cells, changes in cluster attributes, such as mean cluster density, mean cluster circularity and mean cluster area was investigated for 5-HT_2_ receptor clusters. A significant increase in the mean cluster density of 5-HT_2_ receptor clusters at 5 mpi compared to the cluster densities at 0 mpi was observed (Figure 6). Such trends were not observed for DRD2 and Dendra2 samples. Furthermore, to confirm that the increase in the cluster density was due to JCPyV, RDF analyses were performed to measure the probability distribution of molecules in receptor clusters. Results show a significant increase in the aggregation of JCPyV particles within and adjacent to the 5-HT_2_ receptor clusters in infected cells at 5 mpi, with no significant increase observed in the control sample (Figure 7). These findings suggest that JCPyV aggregation in the cluster of 5-HT_2_ receptors might be responsible for the increase in receptor cluster density which eventually leads to endocytosis of the virus-receptor complex. Previous studies established that JCPyV utilizes clathrin-mediated endocytosis for internalization and JCPyV co-localizes with clathrin at 5 mpi [67,110]. Furthermore, the 5-HT_2A_ receptor takes 2–10 min to internalize during the receptor recycling process [111]. These findings suggest that JCPyV-5-HT_2_ receptor complexes may be endocytosed between 5 and 15 mpi; subsequently, as the higher density clusters are presumably internalized, the mean cluster density of 5-HT_2_ receptor clusters and the density of JCPyV particles within the 5-HT_2_ receptor clusters decreases (Figure 8).

Plasma membrane receptors form clusters in dynamic nano-domains on the cell surface that regulate ligand sensitivity to finetune signaling efficiency and control protein interactions [2], examples including GPCRs [3], immune-cell receptors [1], as well as receptors hijacked by microbial toxins [4] or viruses [5]. As human diseases are correlated to the aberrations in the distribution of membrane-bound receptors and/or their activation, it is important to characterize and understand the mechanisms underlying the dynamic rearrangement and clustering of receptors, as it may aid in developing novel strategies for therapeutic treatment of diseases [8]. In the past two decades, several studies have emphasized the importance of the spatial localization of GPCRs to the response of specific signaling pathways [6,7]. Recent studies demonstrate how the organization of GPCRs at the plasma membrane in intracellular membranes provides platforms for distinctive signaling, which are critical for key physiological functions [6]. 5-HT receptors, like other GPCRs, are known to cluster in membrane domains where they interact with other proteins, such as scaffolding protein postsynaptic density 95 (PSD95) and caveolin-1, which are regulated for the internalization process [112,113]. Increased use of super-resolution microscopy techniques has been helpful for the nanoscale study of GPCRs, including the formation of homo- and hetero-oligomers and other kinds of clusters [9,10]. While 5-HTRs are capable of homo- and hetero-oligomerization [114,115,116], the current study focused only on the expression of a single 5-HT_2_R subtype. Further, HEK293A cells utilized in this study, express very low levels of 5-HT_2_Rs [66,89], and thus it is not expected that expression of endogenous 5-HT_2_Rs would have driven significant homo- or hetero-oligomerization to impact the findings reported herein.

DRD2 was included in this study because it is a GPCR but is not supportive of JCPyV infection (Figure S2) [65]. However, we observed colocalization of JCPyV-647 with DRD2-Dendra2 at surface levels comparable to the 5-HT_2_Rs at all three timepoints (Figure 3), yet DRD2 did not enhance viral entry into the cell (Figure 5). Furthermore, while the cluster properties and colocalization of JCPyV with 5-HT_2_Rs changed dynamically with time, these trends were not observed for DRD2. These data suggest that JCPyV localization with DRD2 could be either due to a specific interaction between DRD2 and JCPyV or due to heterodimerization between the GPCRs, DRD2 and 5-HT_2_R [117], that serve as a target for JCPyV during infection [117,118]. An alternative explanation is that DRD2-5-HT_2_R heterodimers may act as a decoy receptor to limit host-cell infection.

Furthermore, our results indicate that there are alterations in cluster properties of 5-HT_2_Rs upon infection. Using RDF and cluster analysis, changes in cluster properties and significant increases in the density of JCPyV particles adjacent to or within receptor clusters were observed at 5 mpi for 5-HT_2_Rs, a time consistent with endocytosis [111], while JCPyV particle density was not significantly increased in DRD2 clusters (Figure 7). These data are supported by previous work [68] and suggest that aggregation of the virus at 5 mpi within receptor clusters may be due to viral endocytosis via 5-HT_2_Rs, while JCPyV interactions with 5-HT_2_Rs or DRD2 at 0 and 15 mpi could activate signaling events necessary for entry and infection. Additional studies would be required to further define whether DRD2 plays a specific role in JCPyV infection and to define whether GPCR-induced signals orchestrate JCPyV infection.

Understanding the spatial arrangement and dynamics of viral and host cell components in cellular membranes is essential to elucidating virus-host interactions and their functional consequences [1]. Insight into the JCPyV attachment and entry processes can provide valuable knowledge for the development of treatments for the fatal disease PML and other viral-mediated diseases. With advancements in optics, spectroscopy, and nanoscale surface patterning, novel methodologies have brought a new perspective in understanding how cells respond to the environment and can help clarify mechanisms of molecular interaction and signaling that are not feasible by other methods [2].

## Figures and Tables

**Table 1:** Summary of JCPyV-647 Manders’ colocalization coefficient with all 5-HT_2_R subtypes in comparison to DRD2 sample at 5 mpi and 15 mpi. JCPyV-Alexa647 colocalization with all subtypes of 5-HT_2_R is significant at 5 mpi when compared to DRD2 receptor. Data were gathered using widefield FPALM imaging.

Time Point	Cell Line	Manders’ Coefficient	Standard Error of Mean	% Difference with DRD2 Dendra2 (± Error)	*p* Value
5 mpi	2A Dendra2	0.766	0.020	58 (±6)	<0.001
	2B Dendra2	0.703	0.024	45 (±6)	<0.001
	2C Dendra2	0.823	0.022	69 (±6)	<0.001
	DRD2 Dendra2	0.486	0.026		
15 mpi	2A Dendra2	0.810	0.025	0.9 (±5)	>0.05
	2B Dendra2	0.756	0.024	−6 (±5)	>0.05
	2C Dendra2	0.886	0.016	10 (±4)	>0.05
	DRD2 Dendra2	0.803	0.027		

**Table 2:** Summary of percent difference of JCPyV-647 Manders’ colocalization coefficient with all 5- HT_2_R subtypes and DRD2 at 5 mpi and 15 mpi. Each row shows the comparison of the sample at the start of row (left) with samples in individual columns and expresses the percent increase/decrease in the sample on the left to that of the one in the column.

	Percent Difference	2A Dendra2	2B Dendra2	2C Dendra2	DRD2 Dendra2
5 mpi	2A Dendra2		9% (±4%)	−7% (±4%)	58% (±6%)
	2B Dendra2	−8% (±4%)		−14% (±4%)	45% (±6%)
	2C Dendra2	7% (±4%)	17% (±4%)		69% (±6%)
	DRD2 Dendra2	−36% (±6%)	−31% (±6%)	−41% (±6%)	
15 mpi	2A Dendra2		7% (±4%)	−9% (±4%)	0.9% (±5%)
	2B Dendra2	−7% (±4%)		−14% (±4%)	−6% (±5%)
	2C Dendra2	9% (±5%)	17% (±4%)		10% (±4%)
	DRD2 Dendra2	−1% (± 5%)	6% (±5%)	−9% (±4%)	

## Data Availability

Not applicable.

## Supplementary Materials

The following supporting information can be downloaded at: https://www.mdpi.com/article/10.3390/v14122597/s1, Figure S1: Generation of 5-HT_2_R constructs.; Figure S2: Infectivity assay for 5-HT_2_R-Dendra2 expressing HEK293A cells.
