# Supplementary material for: Dynamics and Patterning of 5-Hydroxytryptamine 2 Subtype Receptors in JC Polyomavirus Entry
Source: Viruses. 2022 Nov 22;14(12):2597. doi: 10.3390/v14122597 (PMC9782046; doi:10.3390/v14122597)
Supplement: Supplementary file 1 [file viruses-14-02597-s001.zip › viruses-2043611-supplementary.pdf]

Mehmood et al. Supplementary Figures

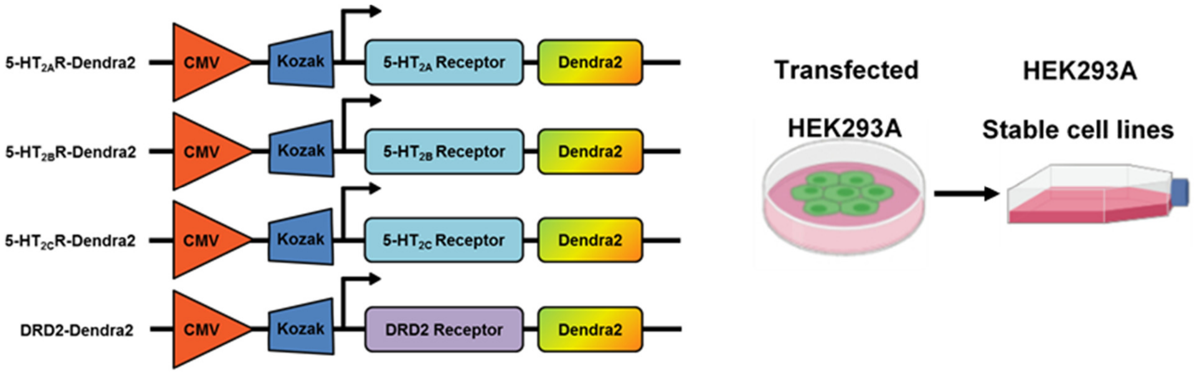

Figure S1. Generation of 5-HT$_2$R constructs. Schematic configuration of 5-HT$_2$R (5-HT$_{2A}$, 5-HT$_{2B}$ and 5-HT$_{2C}$) and DRD2-Dendra2 constructs used for generating stable HEK293A stable cell lines. Schematic created in BioRender.

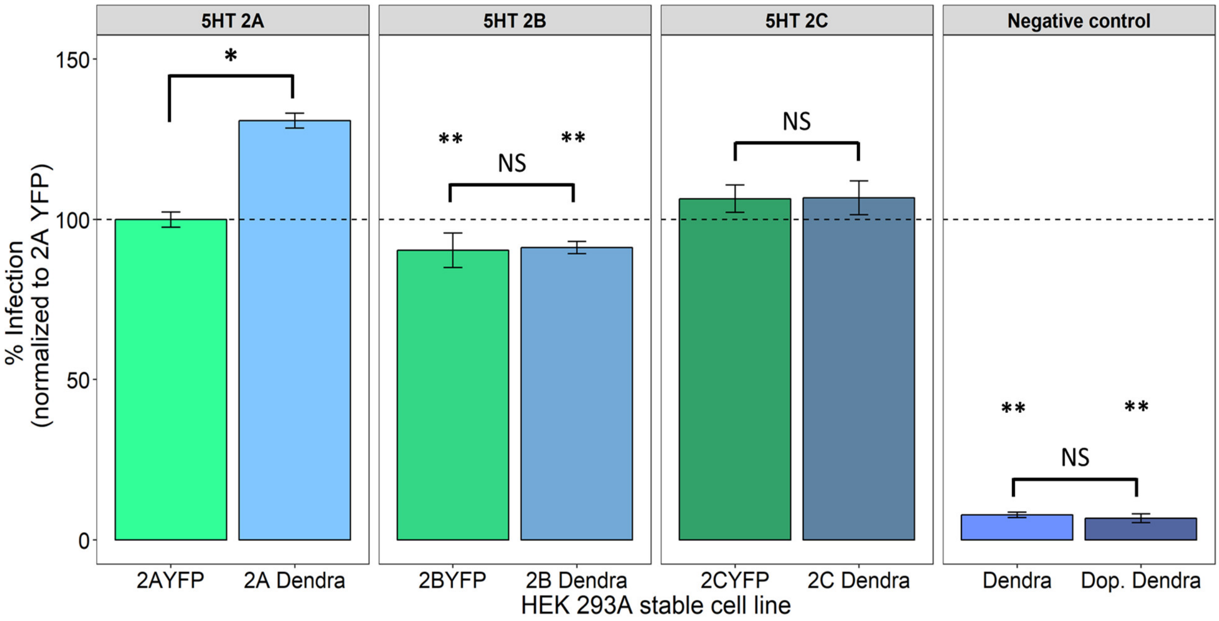

Figure S2. Infectivity assay for 5-HT$_2$R-Dendra2 expressing HEK293A cells. HEK293A stable cell lines expressing 5-HT$_2$R-Dendra2 constructs facilitates JCPyV infection. HEK293A cells, expressing 5-HT$_2$R-YFP (5-HT$_{2A}$R-YFP, 5-HT$_{2B}$R-YFP and 5-HT$_{2C}$R-YFP) and 5-HT$_2$R-Dendra2 constructs, were infected with JCPyV (MOI= 2 FFU/cell) at 37°C for 48 hrs. Cells were fixed and stained for JCPyV T-Antigen (T-Ag) to measure infectivity. Infection was scored by the presence of nuclear T-Ag at 10x magnification for five fields of view per sample for triplicate samples. Data represent the average of three independent samples. Error bars represent SD. **, $p < 0.05$; NS, not significant; **, $p < 0.05$ vs 5-HT$_{2A}$R-YFP.*
